# Speech Understanding and Sound Localization with a New Nonimplantable Wearing Option for Baha

**DOI:** 10.1155/2018/5264124

**Published:** 2018-09-25

**Authors:** Tom Gawliczek, Wilhelm Wimmer, Fabio Munzinger, Marco Caversaccio, Martin Kompis

**Affiliations:** ^1^Hearing Research Laboratory, ARTORG Center for Biomedical Engineering Research, University of Bern, 3008 Bern, Switzerland; ^2^Department of ENT, Head and Neck Surgery, Inselspital, University of Bern, 3010 Bern, Switzerland

## Abstract

**Objective:**

To measure the audiological benefit of the Baha SoundArc, a recently introduced nonimplantable wearing option for bone conduction sound processor, and to compare it with the known softband wearing option in subjects with normal cochlear function and a purely conductive bilateral hearing loss.

**Methods:**

Both ears of 15 normal hearing subjects were occluded for the time of the measurement, yielding an average unaided threshold of 49 dB HL (0.5 – 4 kHz). Soundfield thresholds, speech understanding in quiet and in noise, and sound localization were measured in unaided conditions and with 1 or 2 Baha 5 sound processors mounted on either a softband or a SoundArc device.

**Results:**

Soundfield thresholds and speech reception thresholds were improved by 19.5 to 24.8 dB (p<.001), when compared to the unaided condition. Speech reception thresholds in noise were improved by 3.7 to 4.7 dB (p<.001). Using 2 sound processors rather than one improved speech understanding in noise for speech from the direction of the 2^nd^ device and sound localization error by 23° to 28°. No statistically significant difference was found between the SoundArc and the softband wearing options in any of the tests.

**Conclusions:**

Bone conduction sound processor mounted on a SoundArc or on a softband resulted in considerable improvements in hearing and speech understanding in subjects with a simulated, purely conductive, and bilateral hearing loss. No significant difference between the 2 wearing options was found. Using 2 sound processors improves sound localization and speech understanding in noise in certain spatial settings.

## 1. Introduction

Bone anchored hearing systems (BAHS) are an established method for the treatment of conductive and mixed hearing loss [1–3] and, more recently, also for the treatment of single sided sensorineural deafness [4–7]. BAHS consist of a skin penetrating abutment mounted on an osseointegrated titanium implant located behind the ear. When in use, an external sound processor is snapped to the abutment by the user. Sound is transmitted from the transducer within the sound processor, via the implant to the skull and ultimately by bone conduction to the inner ears of the user [8].

The audiological benefits of BAHS are well documented and the devices are widely used [2, 3, 5, 6, 9]. Nevertheless, at least 2 drawbacks remain: the need for a surgical intervention and the skin penetrating abutment, with a tendency to low-grade infections around the implant [10, 11]. In response to the latter of these two issues, several transcutaneous systems have been developed so far, e.g., the BAHA Attract® (Cochlear Inc., Mölnlycke, Sweden) [12], the Bonebridge system (MED-EL, GmbH, Innsbruck, Austria) [13], the Sophono™ system (Medtronic, Inc., Fridley, Minnesota, USA) [14], or the bone conduction implant (BCI, not yet commercially available) [15]. While the skin above the implant is ultimately intact after the implantation in these transcutaneous systems [10–15], a surgical intervention is still needed.

Especially in young children, but also in a growing population of older children, adolescents, and even adults, there is a demand for solutions in which users can benefit from the advantages of a BAHS without having to undergo surgery.

Until recently, there have been mainly two different nonsurgical solutions to use BAHS sound processors: headbands and softbands [16]. Headbands consist of a steel spring reminiscent of a diadem, to which a disc with a connector for a BAHS processor is attached. Headbands are often used for temporary preoperative trials but occasionally also for permanent use. Softbands are elastic bands worn around the head and are most frequently used in young children [16].

There are two main disadvantages which are associated with the use of these nonimplantable wearing options. One is the sound attenuation by the skin, which increases with high frequencies and reaches, e.g., approximately 15 dB at 3000 Hz [12, 16]. As a result, these nonimplantable solutions are almost exclusively an option for persons with normal or near-normal cochlear functions, such as children with a purely conductive hearing loss. The second drawback is the limited aesthetic appeal. In our experience, the visibility of headbands and softbands often thwarts the use in older children, who become self-conscious, and also in adults.

**Figure 1:**
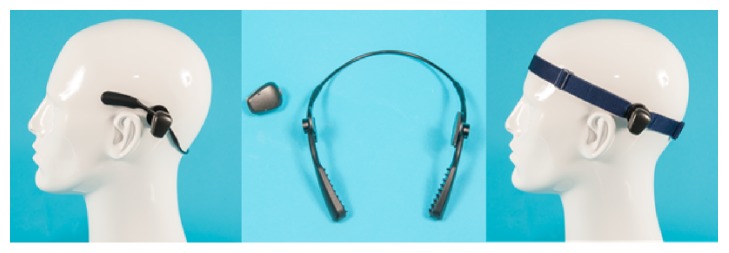
Left: dummy head with a medium power sound processor (Cochlear Baha 5) mounted on a Baha SoundArc. Middle: view of the SoundArc and the sound processor separately. Right: sound processor mounted on a softband.

Recently, a new wearing method for BAHS sound processors without the need for surgery has become available. The Baha SoundArc (Cochlear Inc, Mölnlycke, Sweden) is shown in Figure 1. It is a flexible titanium bow to be worn behind the head, rather than around the head. Similar to the headband and the softband, a disk supporting the sound processor is attached to the side of the device.

From a clinical point of view, an important question in this context is whether the audiologic performance with the BAHS sound processor is not compromised by choosing the new wearing option. To our knowledge, this has not been investigated to date. With this study, we would like to start to fill this gap.

The primary aim of this investigation is therefore to test the audiological benefit of a current BAHS sound processor worn on a SoundArc in terms of soundfield hearing thresholds, speech understanding in quiet, and speech understanding in noise in persons with a purely conductive hearing loss, as this is the population which is expected to benefit most likely from the new option. The second aim of this study is to compare the audiological benefit with the softband, as this is the most frequently used solution, which is already available today. The third aim is to investigate and to quantify the binaural benefit when using two sound processors on a single SoundArc instead of just one.

## 2. Materials and Methods

### 2.1. Ethics

This prospective study was approved by the local ethical committee of Bern (KEK-BE 2017-00642). All tests were performed at the University of Bern in accordance with the declaration of Helsinki.

### 2.2. Study Population and Simulated Conductive Hearing Loss

15 young and normal hearing volunteers (aged 18 to 34 years, 6 women, 9 men) participated in this study. Their air conduction (AC) and bone conduction (BC) thresholds were better than 20 dB HL at all audiometer frequencies between 250 Hz and 6000 Hz. A bilateral conductive hearing loss was simulated for the duration of the tests by blocking both ears with a combination of ear plugs (E-A-Rsoft™, 3M, Berkshire, UK) and an additional filling of the remaining volume within the pinna with silicon mould material (Otoform Ak®, Dreve Otoplastik GmbH, Unna, Germany).

The rationale for the choice of normal hearing subjects and a simulated conductive hearing loss rather than real patients is as follows. All currently available nonsurgical wearing options for BAHS sound processors are known to introduce a considerable high frequency skin attenuation at higher frequencies [12, 16]. As a consequence, they are used predominantly in patients with normal or almost normal inner ear function. At our center, the most important group of users are children, especially young ones. As our test protocol is lengthy and taxing for the subjects, children could not participate as subjects in this study. With normal hearing, adult volunteers with a temporary, simulated conductive hearing loss, the audiological properties of the main target group can be closely emulated and all tests required can be performed. As an additional advantage, the study group is audiologically homogenous and, as all subjects have a* bilateral* simulated hearing loss, the binaural benefit can be easily measured. Further implications of this choice of subjects are discussed in Section 4.3.

A combination of earplugs and silicon mould was used to simulate conductive hearing loss, as either of these blocking methods by itself did not yield a sufficiently high sound attenuation in preliminary tests and circumaural hearing protectors cannot be used together with either the softband or a SoundArc wearing option, because of their size and spatial interference.

### 2.3. Study Protocol

After otoscopy and pure tone audiometry, both ears of each subject were blocked as described above. Then all participants underwent a total of 5 series of measurements, one in each of the following 5 conditions: (i) unaided, (ii) aided with one BAHS sound processor mounted on a SoundArc, (iii) aided with 2 BAHS sound processors on a single SoundArc, (iv) aided with one BAHS sound processor on a softband, and (v) aided with 2 BAHS sound processors on a single softband. The order of these 5 conditions was varied systematically between subjects to minimize the effects of learning or fatigue.

The following measurements were performed in each of these conditions: the sound field thresholds were measured using narrow-band noise at 0.25, 0.5, 1, 2, 3, 4, and 6 kHz. The speech reception threshold (SRT) in quiet, i.e., the presentation level required for 50% word understanding, was measured using German two-digit numbers from the Swiss Version of the Freiburger Test [17]. Speech understanding for monosyllabic words from the same Freiburger test was measured at 50 and 65 dB SPL. In all of these tests, the loudspeaker was placed in front of the subject at a distance of 1 m.

**Figure 2:**
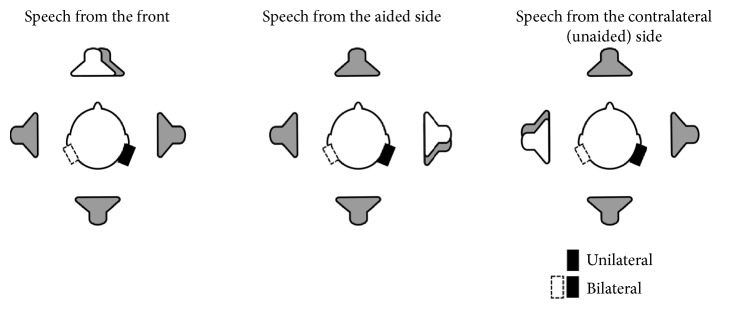
Schematic representation of the spatial settings used for the measurements of the speech reception threshold (SRT) in noise. Uncorrelated noise was emitted by 4 loudspeakers (grey speakers) to create an approximated diffuse noise field. Sentences (white speaker) were presented either from the front, from the side ipsilateral to the sound processor, or contralateral to it.

The SRT in noise, i.e., signal-to-noise ratio required for 50% speech understanding in noise, was measured using the adaptive OLSA test [18]. This test uses an adaptive test procedure and consists of 40 lists of 30 test sentences each and an accompanying noise signal (speech babble) generated by a superposition of all test items [19]. An approximated diffuse noise field was generated by 4 loudspeakers placed around the subjects and emitting uncorrelated noise [19], as shown in Figure 2. The noise was presented continuously at a fixed level of 65 dB SPL at the centre of the setup. One list of test sentences was presented from the loudspeaker from the front, one from the right, and one from the left. Two training lists were administered for each volunteer before the first actual tests started. The results of the training lists were discarded.

Sound localization was measured using 12 loudspeakers spaced 30° apart in a circle with 1 m diameter and centred around the head of the subject. 36 bursts of white noise with a duration of 200 ms were presented in a randomized order and 3 bursts from every loudspeaker at level of 60, 65, and 70 dB SPL, respectively. Subjects indicated from which of the 12 loudspeakers they thought the sound had been presented. Sound localization was measured in all aided conditions.

All of the above measurements were carried out in a sound-attenuated room (6 x 2 x 4 m^3^) with an almost frequency independent reverberation time of approximately 0.14s. JBL Professional Control® 1 PRO loudspeaker (JBL Professional, Northridge, California, USA) was used in all tests.

### 2.4. Sound Processors, Processor Fitting, and Force Measurements

Baha 5 sound processors (Cochlear Inc., Sweden) were used in all aided conditions. They were programmed for each subject and for each of the test conditions separately, following the manufacturer's recommended fitting procedure for conductive hearing loss, using the most recent fitting software (Cochlear BAHA Fitting Software v4.45, Cochlear Inc.) and BC-direct measurements. The settings “position compensation”, “automatic sound classifier”, and “adaptive microphone directionality” were selected.

Baha SoundArcs and softbands were fitted individually for each subject according to the instructions provided by the manufacturer with the products. The force with which the disc with the attached sound processor was pressed to the head was measured 3 times in each subject and for each mounting method using a spring balance (Pesola type Medio-Line 40003, Schindellegi, Switzerland) and the results were then averaged.

### 2.5. Statistical Analysis

To assess the treatment effects, linear mixed-effects models were implemented for each outcome measure. The treatment condition was included as fixed effect (i.e., unaided, SoundArc unilateral, SoundArc bilateral, softband unilateral, and softband bilateral). For SRTs in noise, the test situations (speech from the front, speech from the side of the first sound processor, and speech from the contralateral side) were additionally considered as fixed effects. To account for multiple measures, the subject IDs were included as random effects. Post hoc comparisons between the tested wearing options (i.e., SoundArc versus softband) and number of devices used (unilateral versus bilateral) were performed with general linear hypothesis testing using two-tailed tests and Holm correction [20] for multiple testing. The statistic environment “R” was used for all calculations (R Core Team 2017, ver. 3.4.1 with the “lme4” and “multcomp” packages). The Wilcoxon matched-pairs signed-rank test was used for the comparison between the wearing options in terms of force.

## 3. Results

### 3.1. Aided versus Unaided Hearing Thresholds

**Figure 3:**
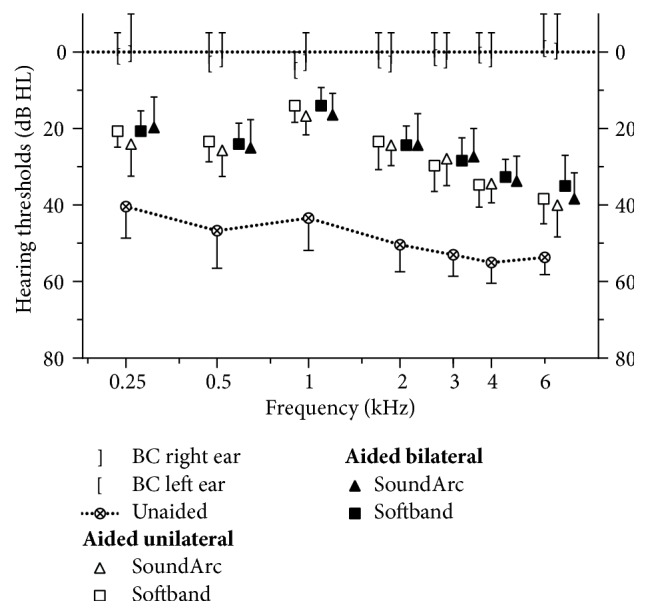
Aided and unaided sound field threshold measured with narrow-band noise. Mean values and standard deviations are shown. See Table 1 for statistical analysis. Mean bone conduction (BC) thresholds of all subjects are shown for comparison.

Figure 3 shows the mean unaided and aided soundfield hearing thresholds in all conditions considered in this project. When averaged over the frequencies of 0.5, 1, 2, and 4 kHz, the unaided soundfield threshold with both ears occluded lies at 49 dB HL. It is slightly better at higher frequencies. A comparison with the average bone conduction thresholds of the subjects, which are close to the 0 dB line for all frequencies in Figure 3, confirms the efficacy of the method used to block both ears.

Aided soundfield thresholds are significantly better (p<.001) than unaided by 23.6 to 25.1 dB, when averaged over the same 4 frequencies between 0.5 and 4 kHz, as above. Statistically, results do not differ between any of the 4 wearing options (softband or SoundArc, unilateral or bilateral) significantly, as shown in detail in Table 1.

### 3.2. Speech Understanding in Quiet

**Figure 4:**
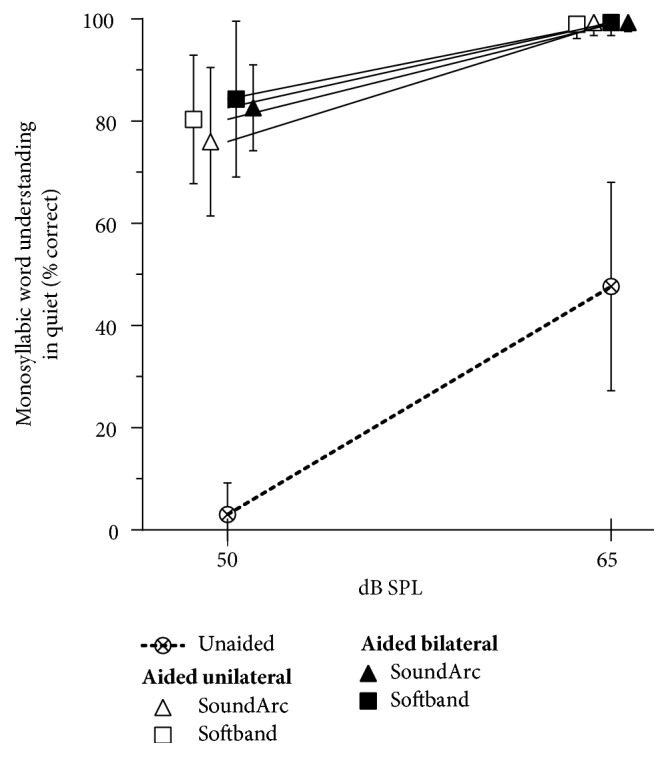
Aided and unaided speech recognition scores for monosyllabic words. Mean values and standard deviations are shown. Symbols and error bars are shifted horizontally for a better visual discrimination between the measurements.

Figure 4 shows the word recognition scores for monosyllabic words in quiet. At 50 dB SPL, statistically highly significant (p < .001) improvements of 73% (SoundArc; unilateral) to 81% (softband, bilateral) are found, when compared with the unaided situation. At 65 dB, the average improvements lie between 51% and 52% (p <.001 for all situations). At 65 dB SPL a ceiling effect is apparent; i.e., speech recognition is almost perfect in all aided conditions. No statistically significant differences between any of the wearing options in the aided conditions can be found at either of the 2 presentation levels.

**Figure 5:**
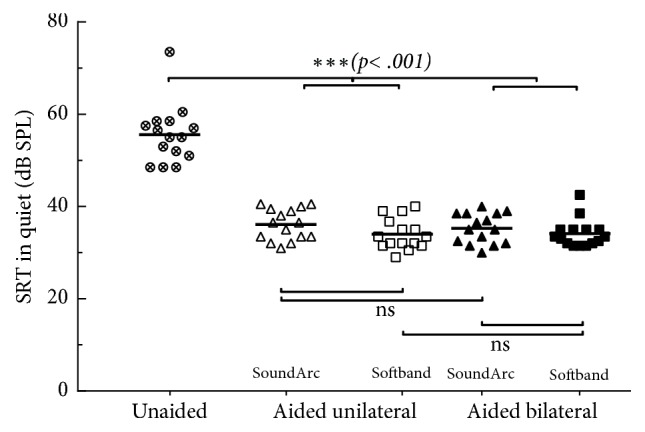
Speech reception thresholds (SRT) in quiet, i.e., presentation levels required for 50% understanding of 2-digit numbers. Individual results (symbols) and mean values (horizontal lines) are shown. Details of the statistical analyses are given in Table 2.

Figure 5 and Table 2 summarize the results of the measurement of the speech reception thresholds in quiet in dB SPL. The average improvements with the Baha range between 19.5 and 21.7 dB and are statistically highly significant (p<.001); in contrast, the differences between the different wearing options (SoundArc or softband, unilateral or bilateral) are, again, minor (0.2 to 2.2 dB) and not statistically significant (p = .26 to .98, cf. Table 2).

### 3.3. Speech Understanding in Noise

**Figure 6:**
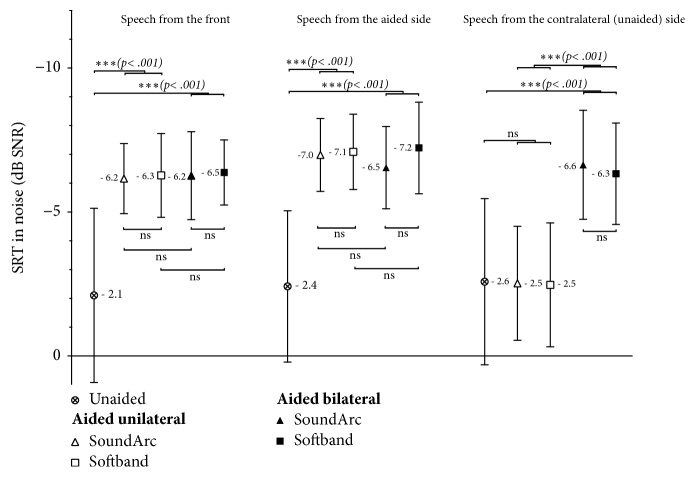
Speech reception thresholds (SRT) in noise. More negative values, i.e., closer to the top of the graph, represent better speech recognition in noise. Mean values across all subjects and standard deviations are shown.

Figure 6 shows the results of the measurements of speech understanding in approximated diffuse noise. Mean SRTs and standard deviations are shown. More negative values denote better speech understanding in noise, and positive values show poorer understanding in noise.

If speech is presented from the front (left hand panel in Figure 6), any of the 4 aided conditions improves the SRT significantly by 4.1 to 4.4 dB. There is no statistically significant difference between SoundArc and softband or between the unilateral and the bilateral use of the aids.

If speech is presented at the side of the Baha (middle panel in Figure 6), SRT are improved, on average, by 4.6 to 4.7 dB. Adding a 2^nd^ device at the other side does not change speech understanding significantly, although the SRT drops slightly (by 0.5 dB) with the SoundArc wearing option.

If a single Baha is used unilaterally and speech is presented from the side of the device (right hand panel in Figure 6), SRTs in noise are not improved. However, if a 2^nd^ Baha is added to the contralateral side, SRT is improved significantly (p<.001) by 3.7 dB with the softband and by 4.0 dB with the SoundArc, respectively.

### 3.4. Sound Localization

**Figure 7:**
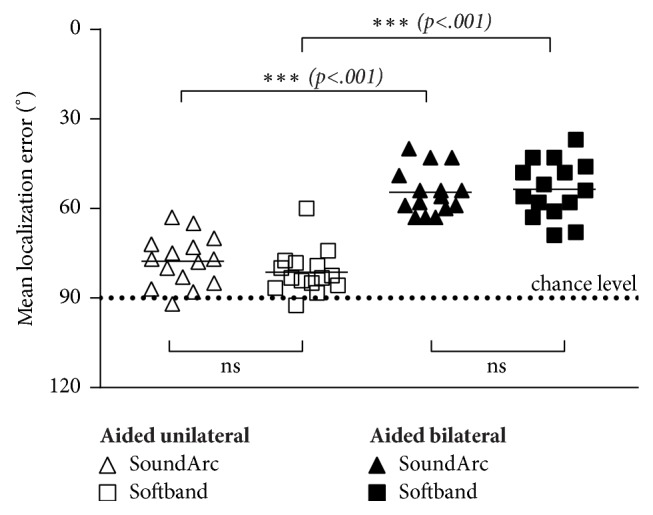
Results of the sound localization measurement with 12 loudspeakers spaced 30° apart. Individual results (symbols) and mean values (lines) are shown. A mean error of 90° corresponds to the chance level expected for guessing.

Figure 7 shows the individual and mean absolute errors for sound localization in all aided conditions. With a single Baha mounted either on a softband or on a SoundArc, average errors are close to 90°, i.e., close to the chance level expected for guessing. Adding a 2^nd^ Baha with either mounting option decreases the localization error by 23° (softband) and 28° (SoundArc), respectively (p< .001). The relatively small differences between both wearing options are not statistically significant in either the unilateral or the bilateral situation.

### 3.5. Force Measurement

The average force needed to lift the holding disc from the skin of the subjects was 1.69 ±0.21 N for the softband and somewhat higher (1.80 ±0.16 N) for the SoundArc. The difference is not statistically significant (p=.178).

## 4. Discussion

### 4.1. Hearing and Speech Understanding

We believe that these results can be adequately summarized into 3 main findings: (1) there is a clear improvement with a Baha mounted on either a SoundArc or a souftband, when compared to the unaided condition, (2) audiologically, no statistically significant difference between the use of a softband and a SoundArc was found, and (3) there is an added audiologic benefit from an additional, 2^nd^ Baha mounted on either a SoundArc or a softband.

The benefit of the aided versus the unaided situation can be seen in the soundfield threshold measurements (Figure 3), but it is also present for speech understanding in quiet (Figures 4 and 5) and in noise (Figure 6). The comparison between sound field thresholds (Table 1) and speech reception threshold in quiet (Table 2) shows that the gain in speech reception thresholds (in dB) is almost as high as the gain in hearing thresholds. This may be a result of the frequency dependency of the aided thresholds, which are best around 1 kHz, and decrease for higher frequencies. The shape of the aided thresholds as a function of frequency in Figure 3 is most probably due to 3 main factors, namely, the resonance frequency of the Baha transducer around 1 kHz, the skin attenuation which increases at higher frequencies [12, 16], and loudness expansion of the sound processors at low input levels.

No statistically significant difference was found between sound processors worn on either a SoundArc or a softband in any of the measurements. This includes sound field thresholds, speech understanding in quiet and in noise, and sound localization. These results seem reasonable, as the same sound processors were used in all tests and the placement as well as the force measured is similar for both mounting methods.

### 4.2. Sound Localization and Additional Benefit from a 2^nd^ Sound Processor

As bone conduction has a much lower sound attenuation across the head than air conduction [21, 22], it is not self-evident that using 2 bone conduction devices can result in a significant audiological benefit when compared to just one device. However, it is known that sound localization is possible with 2 BAHS sound processors mounted on implants, although the localization error is considerably higher than in normal hearing subjects [23]. In this study, it was shown that this is true also for two devices mounted on a softband or on a SoundArc and that sound localization is improved clearly when compared to the use of just one device. What may be even more important for future users, a substantial benefit was found for speech understanding in noise, if the target source was placed at the contralateral side of the first Baha. We have not found a setting, in which the use of a 2^nd^ Baha decreases speech understanding.

As a side effect, the results of the tests in noise shown in Figure 7 can be used as a measure for the reproducibility of our measurements. The measurements for the speech signal from the aided side and from the unaided side (middle and right hand panel in Figure 7) are actually only different, if 1 sound processor is used. For the unaided and the bilaterally aided conditions, the measurements are just repetitions of each other. As can be seen, repeated measurements yield very similar results, as intended.

### 4.3. Relevance

There are two potential issues with the relevance of the presented work: first, the practical importance of the SoundArc and similar devices as an alternative to the already existing softband and second, the applicability of our results to real patients.

Regarding the first of these potential issues, we believe that a new wearing option can be of substantial importance for current and future patients, if it is perceived as attractive by the users. It is known that a significant number of patients chooses not to use or to discontinue the use of bone conduction devices because either a surgical intervention is needed or the nonsurgical wearing option is not aesthetically appealing [7, 24, 25]. It is therefore not surprising that at least one other manufacturer has presented a new nonsurgical alternative to implantable bone conduction devices recently [26]. If, as a result of these and similar new options, more hard-of hearing patients will actually use such devices, we believe that the practical relevance of these new devices is clearly given from the clinical point of view.

All of our tests were performed with normal hearing subjects with bilaterally blocked ears rather than with patients with bilateral conductive hearing loss and with medium-output-power sound processors rather than with power or super-power sound processors.

A number of advantages, but also some limits are associated with this choice. Using young, adult, normal hearing subjects allowed us to study the impact of the devices in subjects with bilaterally normal inner ears and a pure conductive hearing loss. We believe that, in this way, the hearing of children with a purely conductive hearing loss and normal inner ears is adequately simulated. Unlike children, these young adults can easily perform all the rather taxing and time consuming tests required for this study. We expect children and adolescents with a purely conductive hearing loss to become one of the important group of potential users of the SoundArc, as this is an important group for BAHS already today at our centre and at other centres worldwide [27, 28]. Furthermore, the use of normal hearing adults allows us to study a relatively large but homogeneous group of subjects.

As subjects with an additional cochlear hearing loss were not included, we cannot directly draw conclusions from our results on the efficacy of the SoudArc or the softband wearing options for BAHS sound processors in mixed hearing loss or single sided deafness. Nevertheless, we feel that the most important target group for these wearing options is reasonably represented. Future studies may shed more light on the benefit in these groups of users.

As a by-product of this study, we have presented a method which can reliably, safely, and reversibly simulate a considerable conductive hearing loss in the order of magnitude of 45 dB without having to resort to circumaural hearing protectors, which preclude the use of several types of bone conduction hearing devices.

## 5. Conclusions

The results of our study suggest that in subjects with a bilateral conductive hearing loss and a medium power BAHS sound processor mounted on a SoundArc device can benefit from significant improvements in terms of sound field hearing thresholds, speech understanding in quiet, and speech understanding in noise, when compared to the unaided condition. We have not found any significant difference in the audiological performance when compared to the mounting on a softband. The use of an additional 2^nd^ sound processor improved sound localization and speech understanding in noise, if the target speech was presented from the side of the 2^nd^ sound processor.

## Figures and Tables

**Table 1:** Comparison between the unaided and the different unaided soundfield thresholds. All data shown represents average values over the 4 frequencies 0.5, 1, 2, and 4 kHz.

			**Improvement of sound field thresholds (in dB)**			
			Softband		SoundArc	
			Unilateral	Bilateral	Unilateral	Bilateral
Unaided	vs.	aided	**24.8**	**25.1**	**23.6**	**24.0**
			(p <.001)	(p <.001)	(p <.001)	(p <.001)
Unilateral	vs.	bilateral	-	0.3	-	-0.8
				(p = 1)		(p = 1)
Softband	vs.	SoundArc			-1.2 (p = 1)	-1.1
						(p = 1)

**Table 2:** Comparison between the unaided and unaided speech reception thresholds (SRT) in quiet.

			**Improvement of SRT in quiet (in dB)**			
			Softband		SoundArc	
			Unilateral	Bilateral	Unilateral	Bilateral
Unaided	vs.	aided	**21.7**	**21.5**	**19.5**	**20.3**
			(p <.001)	(p <.001)	(p <.001)	(p <.001)
Unilateral	vs.	bilateral	-	0.2	-	-0.8
				(p = .98)		(p = .98)
Softband	vs.	SoundArc	-	-	-2.2	-1.2
					(p = .26)	(p = .98)

## Data Availability

The data used to support the findings of this study are available from the corresponding author upon request.
